# Euphorbia factor L1 suppresses breast cancer liver metastasis via DDR1-mediated immune infiltration

**DOI:** 10.18632/aging.205030

**Published:** 2023-09-14

**Authors:** Dongjing Jiang, XiaoQin Gao, RuLan Tan, Xun Liu, Ye Zhu, Li Zhang

**Affiliations:** 1 Traditional Chinese Medicine and Research Office, Suzhou Health College of Technology, Suzhou 215000, China; 2 Jiangsu Key Laboratory for High Technology Research of TCM Formulae, National and Local Collaborative Engineering Center of Chinese Medicinal Resources Industrialization and Formulae Innovative Medicine and Jiangsu Collaborative Innovation Center of Chinese Medicinal Resources Industrialization, Nanjing University of Chinese Medicine, Nanjing 210023, China

**Keywords:** Euphorbia factor L1, breast cancer, liver metastasis, immune infiltration, DDR1

## Abstract

Euphorbia factor L1 (EFL1), a lathyrane-type diterpenoid from the medicinal herb Euphorbia lathyris L., has been documented to possess various pharmacologic actives. However, the function of EFL1 on breast cancer is not clear. In this study, we explored the effect and mechanism of EFL1 on breast cancer liver metastasis. Female BALB/c mice were subjected to breast cancer-surgical hepatic implantation (SHI) to establish breast cancer liver metastasis model *in vivo*. At 10 days post-surgery, mice were administrated with EFL1 once daily for a total of 2 weeks. Serum AST and ALT activities, abdominal circumference, peritoneal fluid, tumor weight and volume were determined to assess liver and mesenteric re-metastasis of breast cancer. H&E staining was used to observe morphology changes in tumor, liver and small intestine tissues. ELISA was applied to observe inflammatory levels. Tumor DDR1 expression and immune infiltration were determined using western blotting, immunohistochemistry and flow cytometer methods. Our results showed that EFL1 administration improved liver function (AST and ALT activities), ascites, liver metastasis and mesenteric re-metastasis in SHI mice. Also, SHI-induced inflammatory cell infiltration and IL-1β, IL-6, TNF-α generation in ascites were decreased by EFL1 treatment. Mechanism study revealed that EFL1 intervention enhanced the ratios of CD4+ and CD8+ and CD49b+(NK) T lymphocytes and decreased Treg cells through downregulating DDR1 in the tumor of SHI mice. Furthermore, overexpression of DDR1 abolished the anti-liver metastasis effect and pro-immune infiltration action of EFL1 in SHI mice. Together, our findings suggested that EFL1 protects against breast cancer liver metastasis *in vivo* by targeting DDR1-mediated immune infiltration.

## INTRODUCTION

Breast cancer is the most common malignancy in women worldwide in terms of morbidity and mortality, with an estimate of more than 2 million new cases and more than 600,000 deaths in 2018 [1]. Distant metastasis is the leading cause of treatment failure and mortality in breast cancer [2]. The survival rate at 5 years for primary breast cancer is nearly 99%, while it sharply declines to 26% for those with *de novo* metastatic breast cancer [3, 4]. The liver is one of the major metastatic sites of solid tumor metastasis in breast cancer, which occurs in 50–70% of metastatic breast cancer cases [5]. Patients with liver metastasis display hepatic dysfunction, ascites, portal vein thrombosis, nutritional compromise, etc., [5]. Unfortunately, current therapies for breast cancer liver metastasis are primarily hormonal therapy and chemotherapy, which only extend the survival of patients to approximately 18–24 months [3]. Thus, it is urgent to find innovative and effective therapeutic agents for breast cancer liver metastasis.

In the past decade, breast cancer immunotherapies have attracted a great deal of attention for yielding striking responses, but only in a small subset of patients [6]. Effective immunotherapy promotes the killing of cancer cells by activating T cells. This requires the infiltration of cancer-specific T cells such as CD4+, CD8+, and NK1.1+ T lymphocytes [7]. A study found that the occurrence of “immune exclusion” in the tumor microenvironment greatly held back its therapeutic response to immunotherapy [6]. Discoidin domain receptor 1 (DDR1), a collagen receptor and tyrosine kinase, has been identified as a critical element in immune exclusion by working as a central extracellular matrix sensor to modulate cell adhesion [8]. Sun et al. discovered that the extracellular domain of DDR1 regulates isotropic collagen fiber alignment, building up a physical barrier around breast tumors, which leads to T cell exclusion. Furthermore, deletion of DDR1, a collagen activated receptor tyrosine kinase, can promote intratumoral penetration of T cells and suppress tumor growth in mouse models of breast cancer [9]. A study conducted by Han et al. found enhanced DDR1 expression in breast cancer tissues, which negatively affected the prognosis of patients; silencing DDR1 weakened the migrative and invasive abilities of breast cancer, while overexpression of DDR1 facilitated the migration of breast cancer cells [10].

Euphorbia factor L1 (EFL1) is a lathyrane-type diterpenoid isolated from the medicinal herb Euphorbia lathyris L. (Euphorbiaceae) that has a variety of pharmacological activities [11]. EFL1 protects against intestinal barrier impairment and defecation dysfunction in *Caenorhabditis elegans* [12]. EFL1 also decreases osteoclast differentiation by mediating cellular redox status and induces Fas-mediated apoptosis in osteoclasts [13]. Additionally, EFL1 was demonstrated to enhance the efficacy of conventional chemotherapeutic agents in multidrug resistant K562/ADR cells over-expressing ABCB1 [14]. However, the effect of EFL1 on breast cancer remains unclear.

In the present study, we performed breast cancer-surgical hepatic implantation (SHI) in female BALB/c mice to assess the effect of EFL1 on breast cancer liver metastasis and explored the potential mechanism.

## RESULTS

### EFL1 suppresses liver metastasis in breast cancer-SHI mice

**Figure 1:**
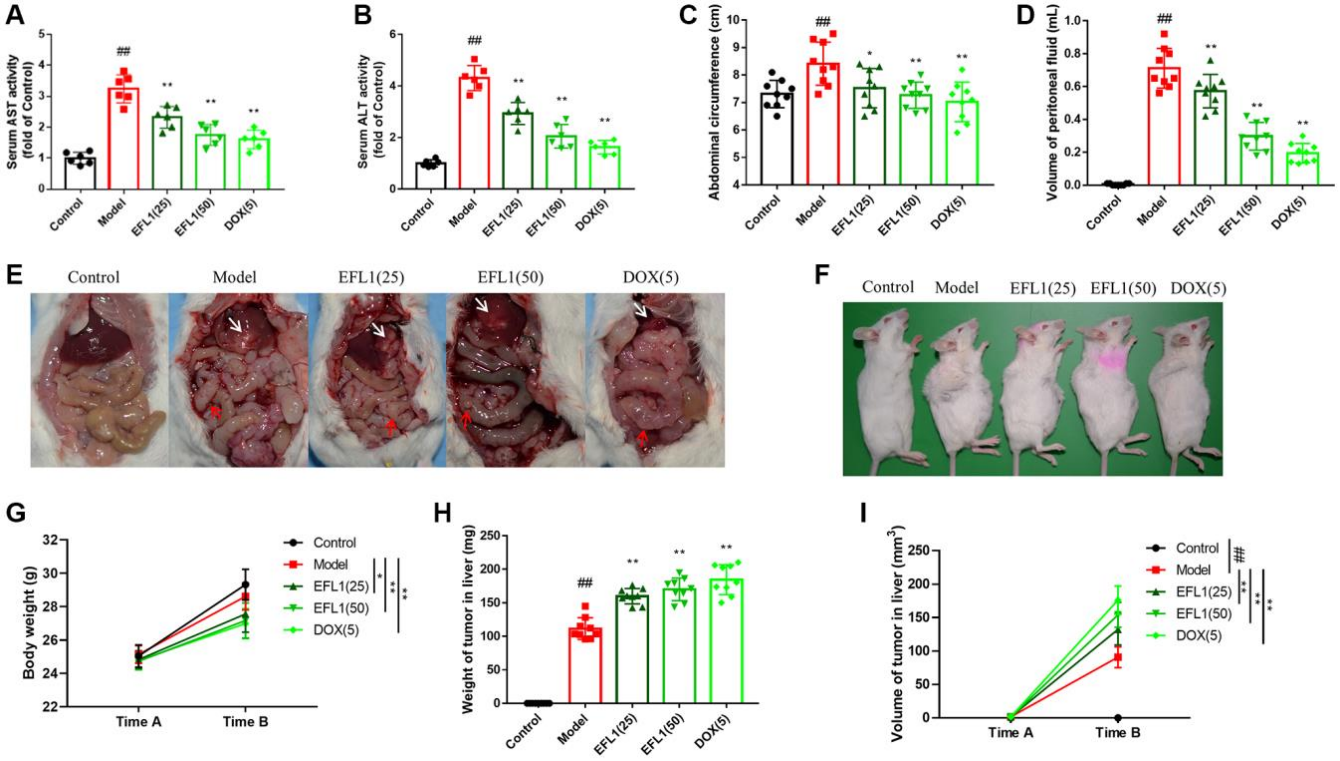
**Protective effect of EFL1 in mice underwent breast cancer-surgical hepatic implantation (SHI).** (**A**, **B**) Serum levels of liver enzymes aspartate aminotransferase. (AST) and alanine aminotransferase (ALT) were determined using commercial kits. *n* = 6. (**C**) Abdominal circumference from surgical hepatic implantation (SHI) mice after 2 weeks of EFL treatment. *n* = 9. (**D**) Peritoneal fluid volume was measured after 2 weeks of EFL1 treatment. *n* = 9. (**E**) Representative images of liver metastasis and mesenteric re-metastasis in SHI mice. White arrows denote SHI tumor and red arrows denote mesenteric re-metastasis. (**F**) Representative images of tumor growth in mice received SHI. (**G**) Body weights recorded right before SHI surgery (Time A) and after 2 weeks of EFL1 administration (Time B). *n* = 9. (**H**) Liver tumor weight at the end of the experiment. *n* = 9. (**I**) Liver tumor volume recorded right before SHI surgery (Time A) and after 2 weeks of EFL1 administration (Time B). *n* = 9. Data were compared using one-way ANOVA with Dunnett’s post hoc (**A**–**D**, **H**) or two-way ANOVA with Tukey’s post hoc (**G**, **I**). ^##^*P* < 0.01 vs. Control; ^*^P < 0.05, ^**^P < 0.01 vs. Model.

We first determined if EFL1 prevents breast cancer liver metastasis using breast cancer (4T1 cell line)-surgical hepatic implantation (SHI) murine models. Compared to control, mice in the model group had enhanced AST and ALT activities, increased abdominal circumference and peritoneal fluid, and evident liver metastasis and mesenteric re-metastasis, suggesting the development of liver metastasis after SHI (Figure 1A–1F). Nonetheless, these were all reversed by EFL1 or doxorubicin (DOX) treatment. Moreover, SHI mice had lower body weight relative to the control group, which might attribute to cancer-induced anorexia-cachexia syndrome. However, EFL1 or DOX intervention further reduced body weight and increased tumor weight and volume (Figure 1G–1I), which might be related to the inhibition of ascites and solid tumor metastasis in SHI mice after EFL1 or DOX intervention (Supplementary Figure 1A–1F).

### EFL1 elicits morphology changes in the tumor, liver and small intestine

**Figure 2:**
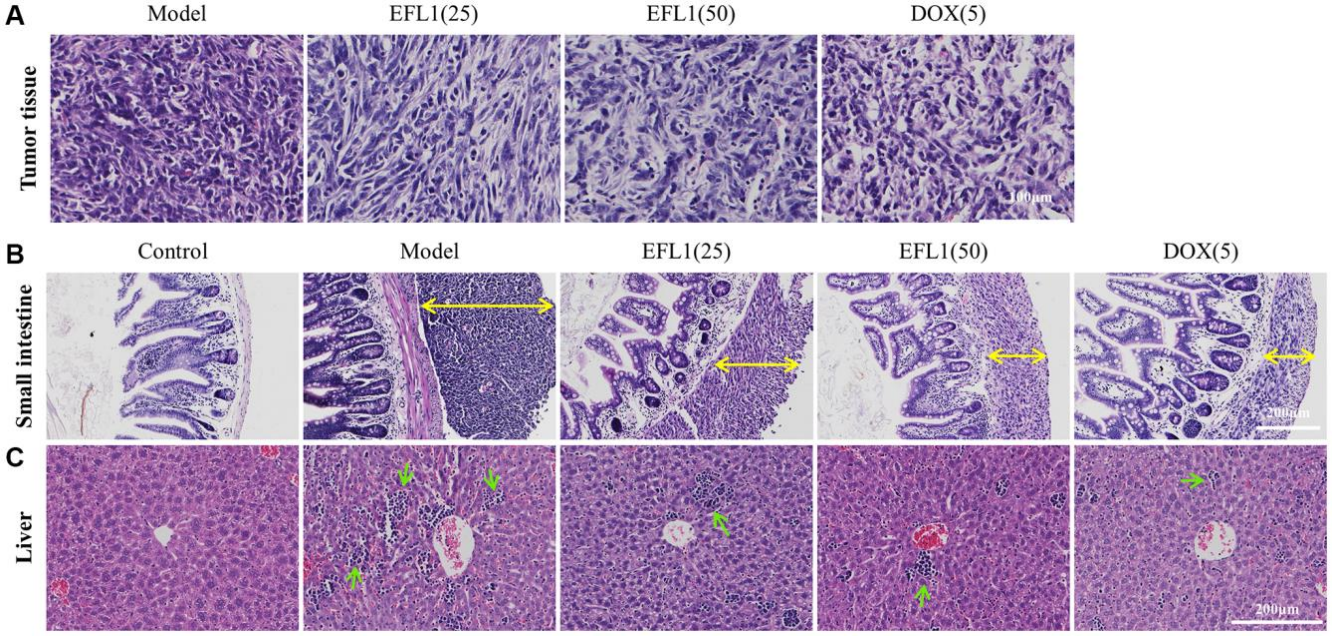
**Representative histology sections stained with H&E.** (**A**) Representative pictures of liver tumor sections. Scale bars: 100 μm. (**B**) Representative pictures of small intestine sections. Yellow double arrows denote tumor size. Scale bars: 200 μm. (**C**) Representative pictures of liver sections. Green arrows denote inflammatory cells. Scale bars: 200 μm.

Results of H&E staining revealed that tumor cells in EFL1 or DOX groups were more scattered than the model group (Figure 2A). Figure 2B shows that tumor-bearing mice had different sizes of tumor tissues in the outer membrane of the small intestines, among which the model group had the largest. EFL1 or DOX administration reduced the migration of tumor cells to small intestine to a certain degree. In Figure 2C, tumor-bearing mice had noticeable inflammatory cell infiltration in liver tissues as compared to control, especially the model group. Nevertheless, EFL1 or DOX intervention ameliorated inflammatory cell infiltration to a certain degree.

### EFL1 decreases proinflammatory cytokine production in ascites

**Figure 3:**
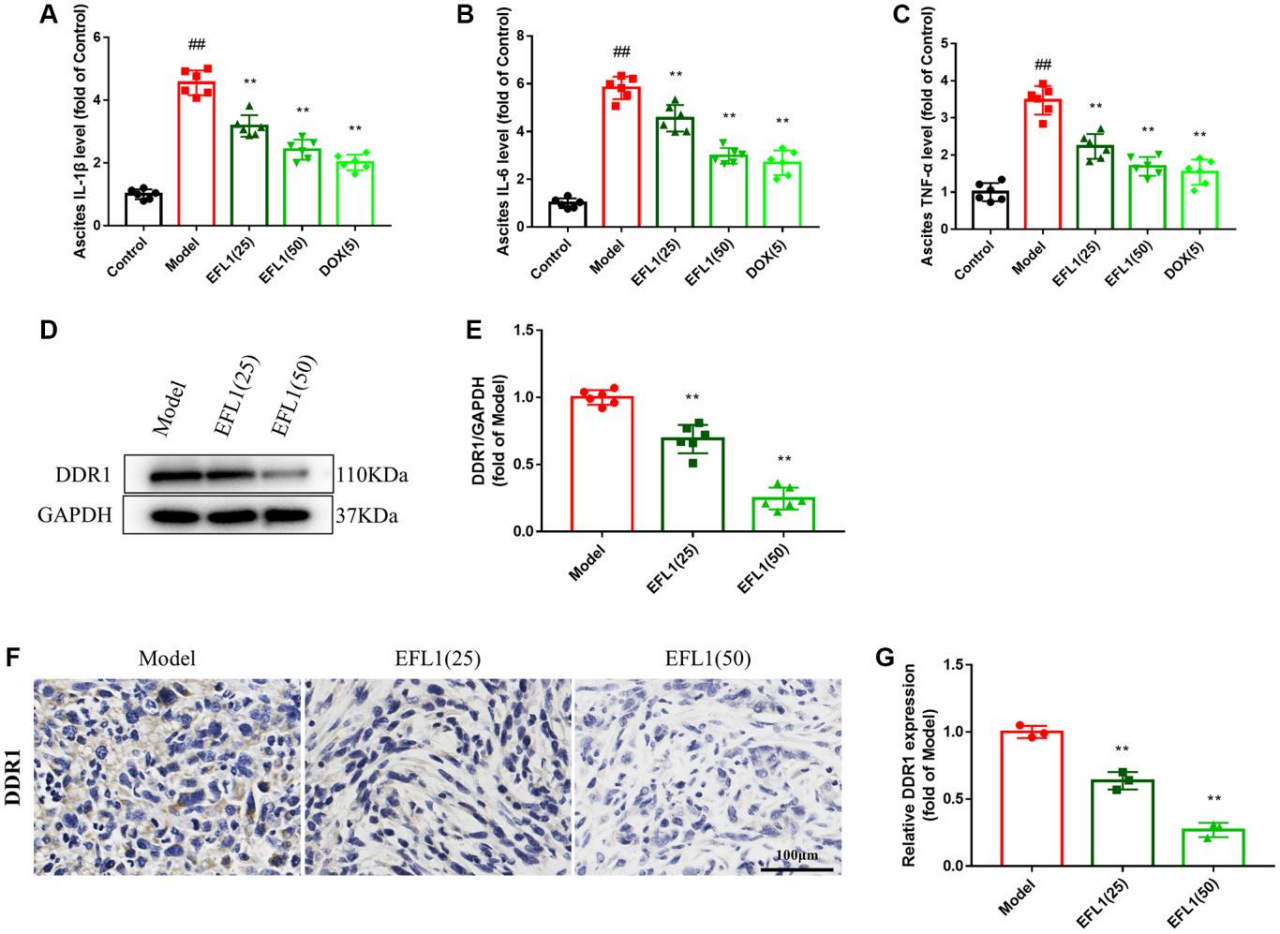
**Effect of EFL1 on DDR1-regulated immune infiltration in SHI mice.** (**A**–**C**) Levels of ascites IL-1β, IL-6, TNF-α were tested by ELISA kits. *n* = 6. (**D**, **E**) Western blot analysis of DDR1 protein expression in tumor tissues. *n* = 6. (**F**, **G**) Relative DDR1 expression in tumor tissues determined by immunohistochemistry. Scale bars: 100 μm. *n* = 3. Data were compared using one-way ANOVA with Dunnett’s post hoc. ^##^*P* < 0.01 vs. Control; ^**^P < 0.01 vs. Model.

ELISA assays were applied to measure the levels of proinflammatory cytokines in ascites. As indicated in Figure 3A–3C, ascites proinflammatory cytokines IL-1β, IL-6, TNF-α were increased in model mice, but were significantly reduced following EFL1 or DOX administration, which demonstrates that EFL1 is able to decrease the generation of proinflammatory cytokines caused by breast cancer liver metastasis.

### EFL1 downregulates DDR1 and promotes immune infiltration in the tumor

**Figure 4:**
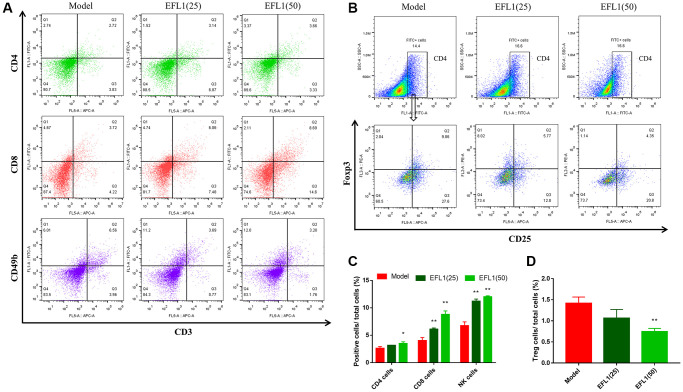
**Measurements of T lymphocytes using flow cytometry.** (**A**, **B**) Representative images of tumor-infiltrating CD4+, CD8+, NK1.1+ T cells and Tregs. (**C**, **D**) Statistical results of tumor-infiltrating CD4+, CD8+, CD49b+ T cells and Tregs. *n* = 3. Data were compared using one-way ANOVA with Dunnett’s post hoc. ^*^P < 0.05, ^**^P < 0.01 vs. Model.

We then explored whether EFL1 represses breast cancer liver metastasis by targeting DDR1-regulated immune infiltration (Supplementary Figure 2). As depicted in Figure 3D–3G and Figure 4A–4D, treatment with EFL1 downregulated DDR1 protein expression and immunoreactivity in SHI mice, leading to the surge of CD4+, CD8+, and CD49b+ (NK) T cells, as well as the reduction of Tregs. This suggested that DDR1-regulated immune infiltration was involved in the inhibitory effect of EFL1 in breast cancer liver metastasis.

### DDR1 overexpression blocks EFL1-induced immune infiltration in the tumor

**Figure 5:**
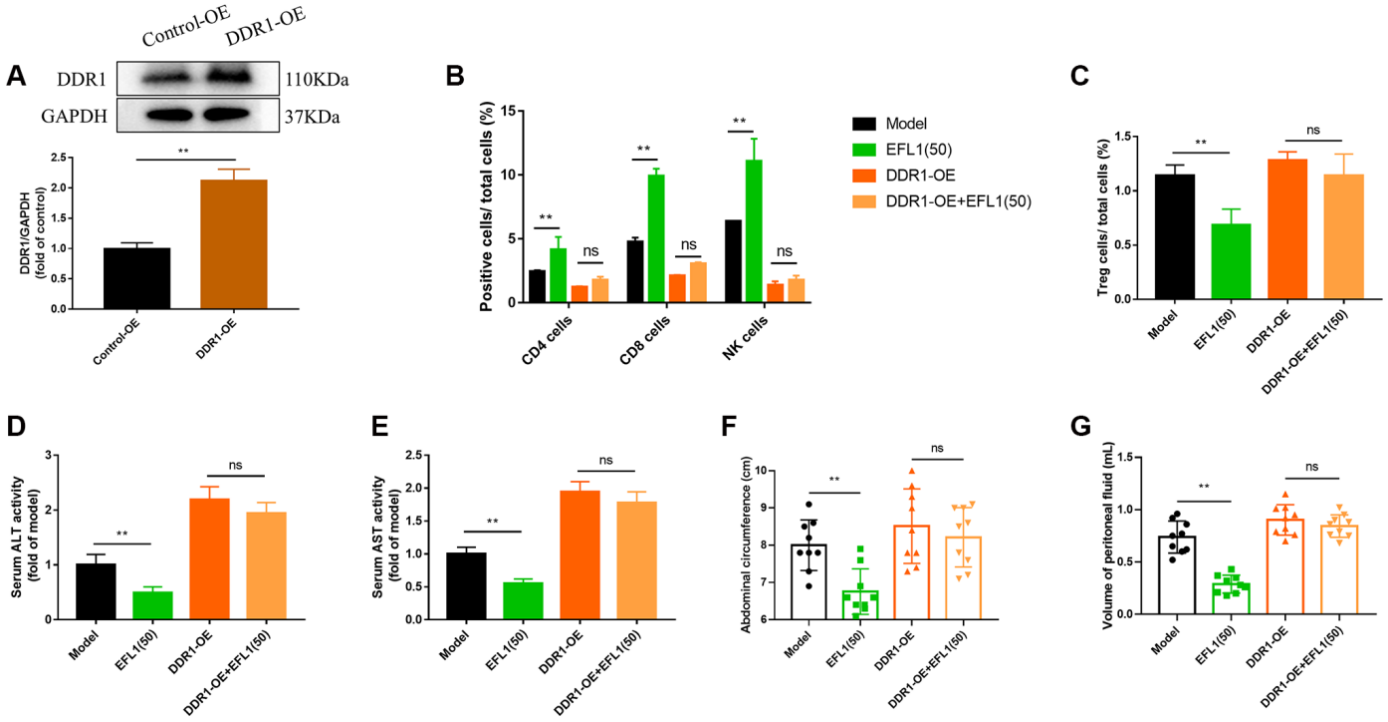
**DDR1 overexpression blocked EFL1-induced beneficial effects in SHI mice.** (**A**) Verification of DDR1 overexpression transfection using western blot analysis. *n* = 6. (**B**, **C**) Tumor-infiltrating CD4+, CD8+, CD49b+ T cells and Tregs were analyzed by flow cytometry. *n* = 3. (**D**, **E**) ALT and ALT activities in mouse serum. *n* = 6. (**F**, **G**) Abdominal circumference and peritoneal fluid volume of mice in each group. *n* = 9. Data were compared using unpaired Student’s *t* test (**A**) or two-way ANOVA with Tukey’s post hoc (**B**–**G**). ^**^P < 0.01. Abbreviation: ns: not significant.

To further investigate the role of DDR1 in EFL1-produced protection against breast cancer liver metastasis, 4T1 cells were transfected with DDR1 overexpressing plasmids before being injected to the mice (Figure 5A). As shown in Figure 5B, 5C, no significant differences were detected in CD4+, CD8+, CD49b+ T cells, and Tregs between the DDR1-OE group and DDR1-OE+EFL1 group, indicating the abolishment of EFL1-produced immune infiltration in SHI mice.

### DDR1 ablates EFL1-induced anti-liver metastasis effects in breast cancer SHI mice

**Figure 6:**
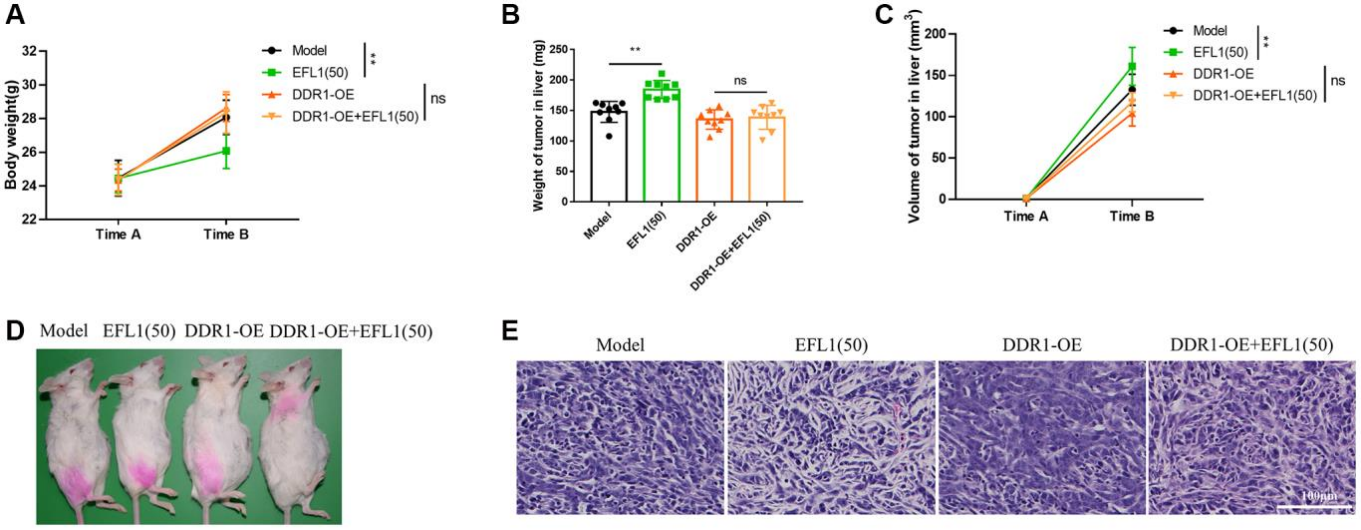
**Body weight and tumor characterization following DDR1 overexpression.** (**A**) Body weights were recorded right before SHI surgery (Time A) and after 2 weeks of EFL1 administration (Time B). *n* = 9. (**B**) Liver tumor weight at the end of the experiment. *n* = 9. (**C**) Liver tumor volume was recorded right before SHI surgery (Time A) and after 2 weeks of EFL1 administration (Time B). *n* = 9. (**D**) Representative images of tumor bearing mice at the end of the experiment. (**E**) Representative histology of H&E stained tumor sections from experimental groups. Data were compared using two-way ANOVA with Tukey’s post hoc. ^**^P < 0.01; Abbreviation: ns: not significant.

As expected, DDR1 overexpression compromised EFL1-induced pharmacological effects on liver dysfunction, ascites, body weight, tumor weight, tumor volume and tumor morphology (Figure 5D–5G and Figure 6A–6E). Together, these data suggested that DDR1 inhibition is required for EFL1 treating breast cancer liver metastasis.

## DISCUSSION

Liver metastasis is a leading cause of morbidity and mortality in cancers, including breast cancer [15]. Thus, there has been strong interest in the development of agents that can effectively prevent liver metastasis. Growing evidence suggests that natural products possess profound anti-liver metastasis property in breast cancer. Crocin, the main bioactive carotenoid of saffron, prevents metastasis of triple-negative breast cancer by interfering with Wnt/β-Catenin pathway in murine models [16]. Compared to the nontreated control group, green tea extract significantly decreased liver metastasis in BALB/c mice bearing 4T1 tumors by 72.6% [17]. Baicalin, a flavonoid compound isolated from the roots of Scutellaria lateriflora Georgi, reduces the number of breast cancer metastatic nodules on the surface of the liver through reversing epithelial-to-mesenchymal transition [18]. Emodin, an active anthraquinone derivative isolated from Rheum palmatum, inhibits liver metastasis of triple negative breast cancer cells in mice by decreasing the secretion of CCL5 in serum [19]. In our work, EFL1 reduced liver metastasis and mesenteric re-metastasis of breast cancer cells, enhanced liver function and mitigated ascites in breast cancer SHI mice, reflecting the potential of EFL1 in treating breast cancer liver metastasis.

Previously, the immune system has been demonstrated to play an important role in the development of cancer [20, 21]. Malignant cells are captured and eliminated by the tumor-infiltrated lymphocytes’ cytotoxic responses [22]. Therefore, current immunotherapeutic methods mainly focus on T lymphocytes, the major effector cells in cellular immunity that produce cytokines in immune responses to mediate inflammation and regulate other types of immune cells [23, 24]. Natural Killer (NK) T cells are the first responders of the immune system and have an inherent ability to recognize and lyse cancerous cells without prior sensitization or antigen presentation [25]. CD8+ T cells suppress tumor proliferation and metastasis by directly recognizing and killing tumor cells via intracellular antigens [22]. CD4+ T cell help has been known to be essential for sustaining CD8+ T cell function [26]. Treg cells expressing the transcription factor Forkhead Box P3 (FOXP3) are immunosuppressive regulators of immune responses that suppress self-reactive T cells and other cells [27]. The crucial effect of T cells in liver metastasis has been documented in previous research. Yu and his colleagues reported that liver-directed radiotherapy stops hepatic siphoning of CD8+ T cells from systemic circulation, reshapes the liver immune microenvironment, and restores immunotherapy efficacy in models of liver metastases [28]. Treatment that decreases Tregs and increases the expression of CD44 in CD4+ and CD8+ T cells, is able to suppress liver metastases and improve the overall survival rate in rectal cancer [29]. Here, we found that EFL1 improved CD4+, CD8+, and CD49b+ T cells, and decreased Tregs, leading to reduction of inflammation and liver metastasis and mesenteric re-metastasis of breast cancer cells, suggesting that T lymphocytes infiltration was implicated in EFL1-conferred protection in breast cancer liver metastasis.

Discoidin domain receptor1 (DDR1) is a tyrosine kinase receptor that is activated by fibrillar collagens and participates in the progression of liver metastasis and breast cancer [30]. Activation of DDR1/STAT3 signaling pathway by extracellular matrix remodeling promotes liver metastatic colonization (e.g., survival, outgrowth and stemness of cancer cells in the metastatic site) in uveal melanoma [31]. NSD2 circular RNA aggravates liver metastasis of colorectal cancer by regulating miR-199b-5p/DDR1/JAG1 axis [32]. DDR1 depletion inhibits cell growth and cell cycle progression and enhances the sensitivity of PIK3CA/AKT1 mutant cells to palbociclib in estrogen receptor (ER)-positive, HER2-negative breast cancer [33]. Vella et al. observed that DDR1 influences metabolic reprogramming in breast cancer cells by cross-talking to the Insulin/IGF system [34]. In addition, tumor DDR1 promotes collagen fiber alignment and contributes to breast cancer development by instigating immune exclusion [9]. Consistent with these studies, our data indicated increased DDR1 expression in breast cancer liver metastasis mice, which was decreased after EFL1 treatment via T lymphocytes infiltration; however, overexpression of DDR1 abolished EFL1-induced anti-liver metastasis function in breast cancer, suggesting that EFL1-induced anti-liver metastasis effect relies on DDR1 inhibition in mice with breast cancer.

## CONCLUSION

In conclusion, our results suggest that EFL1 protects against breast cancer liver metastasis through targeting DDR1-regulated immune infiltration. This study revealed the suppression effect of EFL1 on breast cancer liver metastasis, and highlights the potential role of inhibiting DDR1 in treating breast cancer.

## MATERIALS AND METHODS

### Breast cancer cell lines

Mouse 4T1 breast cancer cell line was obtained from the Cell Bank of the Chinese Academy of Sciences (Shanghai, China) and grown in DMEM media supplemented with 10% fetal bovine serum, 100 U/ml penicillin, and 100 μg/ml streptomycin at 37°C and 5% CO_2_.

### Animals and treatment

Female BALB/c mice aged 4–6 weeks (*n* = 9 per group) were used in animal experiments. Mice were kept under a specific pathogen-free facility with a 12 h:12 h light: dark cycle at 22 ± 1°C. The animal study was reviewed and approved by the Ethics Committee of Nanjing University of Chinese Medicine. This research does not involve human participants.

The liver metastasis of breast cancer modeling was performed as previously described [35]. Briefly, 200 μL of 4T1 cell suspension at a concentration of 2.5 × 10^7^/mL was injected subcutaneously into the back of the scapula of female BALB/c mice. When the tumors reached 1 cm in diameter, they were harvested and cut into approximately a size of 1 mm^3^. For surgical hepatic implantation, mice were anesthetized with 50 mg/kg sodium pentobarbital. After the left lobe of the liver was exposed, the blind end cavity was pierced with a 25G syringe needle. Tumor fragment was inserted followed by bleeding control with compression. Then, the abdominal wall was sutured with a 5-0 silk thread. The body weight and abdominal circumference of mice were recorded weekly, and drug intervention (EFL1: 25, 50 mg/kg, daily gavage; doxorubicin, 5 mg/kg, tri-weekly intraperitoneal injection) started 10 days after the operation for a total of 2 weeks. On the second day of the last administration, all mice were sacrificed and sampling was conducted.

### Cell transfection

To generate DDR1-overexpressed 4T1 cells, lentiviral plasmids carrying the DDR1 gene (Gene ID: NM_007584) were transfected into 4T1 cells at a multiplicity of infection (MOI) of 20 and tilter 1.0 × 10^8^ TU/mL (transfection time, 24 h). 72 h later, cell proteins were extracted and the transfection efficiency was assessed using western blot assay. The DDR1 overexpressing lentivirus was provided by OBiO Technology (Shanghai) Corp., Ltd.

### Biochemical tests

Blood was collected and serum was separated by centrifugation at 4°C, 3000 rpm for 10 min. The concentrations of serum alanine aminotransferase (ALT, C009-3-1) and aspartate aminotransferase (AST, C010-3-1) were detected using commercially available kits (Jiancheng Bioengineering, China) as per the manufacturer’s instructions. The absorbance value was measured by a microplate reader (340PC384, SpectraMax).

The levels of IL-6 (ZC-37988), IL-1β (ZC-37974), TNF-α (ZC-39024) in ascitic fluid were determined using ELISA kits (Zhuocai Biotechnology, China) according to the manufacturer’s instructions.

### Hematoxylin and eosin (H&E) staining and immunohistochemistry

Tissues were fixed in 10% formalin, embedded in paraffin, and sectioned at a thickness of 5 μm. Slices were then stained with hematoxylin and eosin (C0105, Beyotime) following the manufacturer’s protocol and visualized using a light microscope (DM4000B, Leica).

Immunohistochemistry for DDR1 was performed as previously described [36, 37]. Tissue sections were heated with 1X sodium citrate buffer for antigen retrieval in a microwave, incubated with 3% hydrogen peroxide for 10 min to inhibit endogenous peroxidase activity, and blocked in 3% normal goat serum for 1 hour at room temperature. Next, the antibody against DDR1 (ab150506, Abcam, 1:200) was added and incubated overnight at 4°C, followed by the incubation of a secondary antibody (BL003A, Biosharp, 1:1000) at room temperature for 1 h. Sections were then covered with DAB, counterstained with hematoxylin, and mounted. Immunostaining sections were imaged using a light microscope (DM4000B, Leica). Results of immunohistochemistry were quantified by ImageJ software.

### Western blot

Western blotting was conducted following a previously published protocol [38]. In brief, tumor tissues were lysed in RIPA lysis buffer supplemented with 1% PMSF and 2% phosphatase inhibitors. Lysates were centrifuged and protein concentrations were measured using BCA assay. An equal amount of protein was separated by 12% SDS-polyacrylamide gel, and transferred to polyvinylirdenediflouride (PVDF) membranes. After being blocked in 3% BSA for 1 hour, the membranes were incubated with the primary antibodies against DDR1 (CY2924, Abways, 1:1000) and GAPDH (5174, Cell Signaling Technology, 1:5000) followed by the HRP-conjugated anti-rabbit IgG (14708, Cell Signaling Technology, 1:3000). Bands were visualized using an enhanced chemiluminescence detection kit and Tanon 4600 chemiluminescent imaging system, and analyzed by ImageJ.

### Flow cytometry

CD4, CD8 and NK cell staining (surface antigen staining): Fresh tumor tissues were minced and then digested in serum-free medium containing 1 mg/mL collagenase D and 10 U/mL DNase I for 1 hour. After adding serum to stop digestion, cells were transferred to a cell mesh with a pore size of 100 μm and grounded to prepare a single-cell suspension. Cells were then transferred to a 15 mL centrifuge tube, centrifuged at 3000 rpm for 5 minutes, and the supernatant was discarded. Subsequently, cells were resuspended in 50 μL of antibody blocking solution (1:100 anti-mouse CD16/CD32 Fc block) prepared in FACS solution, and incubated at room temperature for 10 minutes. 50 μL of 2× membrane surface antibody mixture prepared in FACS solution was added and incubated at 4°C for 1 hour. After two washes in FACS solution, cells were tested on the flow cytometry machine (CytoFlex, Beckman, USA).

Treg cell staining (intracellular antigen staining): After adding CD4 and CD25 antibodies for staining according to the surface antigen staining protocol above, Foxp3 Fix/Perm Buffer (4×, 421401, Biolegend) was added for fixing and breaking membrane. Cells were then incubated with Foxp3 antibody and tested on the flow cytometry machine. The antibodies used in this study were CD3 (100236, Biolegend, 1:100), CD4 (100406, Biolegend, 1:200), CD8 (100706, Biolegend, 1:100), CD49b (108913, Biolegend, 1:100), CD25 (101910, Biolegend, 1:100), Foxp3 (320008, Biolegend, 1:100).

### Statistical analysis

Data is expressed as mean ± standard deviation (SD) and analyzed using GraphPad Prism 7 software. Three and more groups of data were compared using one-way or two-way analysis of variance (ANOVA), followed by Dunnet or Tukey’s post test. Two groups of data were compared using unpaired Student’s *t* test. Only *p* values < 0.05 were considered significant.

### Data availability

The original contributions presented in the study are included in the Article/Supplementary Materials; further inquiries can be directed to the corresponding authors.

## Supplementary Materials

Supplementary Materials

Supplementary Figures

## References

[1] Chew NJ, Lim Kam Sian TCC, Nguyen EV, Shin SY, Yang J, Hui MN, Deng N, McLean CA, Welm AL, Lim E, Gregory P, Nottle T, Lang T, et al. Evaluation of FGFR targeting in breast cancer through interrogation of patient-derived models. Breast Cancer Res. 2021; 23:82. 10.1186/s13058-021-01461-4 34344433 PMC8336364

[2] Liu H, Li X, Li H, Feng L, Sun G, Sun G, Wu L, Hu Y, Liu L, Wang H. Potential molecular mechanisms and clinical progress in liver metastasis of breast cancer. Biomed Pharmacother. 2022; 149:112824. 10.1016/j.biopha.2022.112824 35306430

[3] Wang Z, Yang L, Wu P, Li X, Tang Y, Ou X, Zhang Y, Xiao X, Wang J, Tang H. The circROBO1/KLF5/FUS feedback loop regulates the liver metastasis of breast cancer by inhibiting the selective autophagy of afadin. Mol Cancer. 2022; 21:29. 10.1186/s12943-022-01498-9 35073911 PMC8785480

[4] Ji L, Cheng L, Zhu X, Gao Y, Fan L, Wang Z. Risk and prognostic factors of breast cancer with liver metastases. BMC Cancer. 2021; 21:238. 10.1186/s12885-021-07968-5 33676449 PMC7937288

[5] Rashid NS, Grible JM, Clevenger CV, Harrell JC. Breast cancer liver metastasis: current and future treatment approaches. Clin Exp Metastasis. 2021; 38:263–77. 10.1007/s10585-021-10080-4 33675501 PMC8211035

[6] Fang W, Zhou T, Shi H, Yao M, Zhang D, Qian H, Zeng Q, Wang Y, Jin F, Chai C, Chen T. Progranulin induces immune escape in breast cancer via up-regulating PD-L1 expression on tumor-associated macrophages (TAMs) and promoting CD8^+^ T cell exclusion. J Exp Clin Cancer Res. 2021; 40:4. 10.1186/s13046-020-01786-6 33390170 PMC7780622

[7] Joyce JA, Fearon DT. T cell exclusion, immune privilege, and the tumor microenvironment. Science. 2015; 348:74–80. 10.1126/science.aaa6204 25838376

[8] Duan X, Xu X, Zhang Y, Gao Y, Zhou J, Li J. DDR1 functions as an immune negative factor in colorectal cancer by regulating tumor-infiltrating T cells through IL-18. Cancer Sci. 2022; 113:3672–85. 10.1111/cas.15533 35969377 PMC9633303

[9] Sun X, Wu B, Chiang HC, Deng H, Zhang X, Xiong W, Liu J, Rozeboom AM, Harris BT, Blommaert E, Gomez A, Garcia RE, Zhou Y, et al. Tumour DDR1 promotes collagen fibre alignment to instigate immune exclusion. Nature. 2021; 599:673–8. 10.1038/s41586-021-04057-2 34732895 PMC8839149

[10] Han Q, Xiao F, Ma L, Zhou J, Wang L, Cheng H, Zhu J, Yao F, Lyu J, Du L. DDR1 promotes migration and invasion of breast cancer by modulating the Src-FAK signaling. Neoplasma. 2022; 69:1154–64. 10.4149/neo_2022_220316N289 35818965

[11] Zhu A, Sun Y, Zhong Q, Yang J, Zhang T, Zhao J, Wang Q. Effect of euphorbia factor L1 on oxidative stress, apoptosis, and autophagy in human gastric epithelial cells. Phytomedicine. 2019; 64:152929. 10.1016/j.phymed.2019.152929 31454650

[12] Zhu A, Ji Z, Zhao J, Zhang W, Sun Y, Zhang T, Gao S, Li G, Wang Q. Effect of Euphorbia factor L1 on intestinal barrier impairment and defecation dysfunction in Caenorhabditis elegans. Phytomedicine. 2019; 65:153102. 10.1016/j.phymed.2019.153102 31654989

[13] Hong SE, Lee J, Seo DH, In Lee H, Ri Park D, Lee GR, Jo YJ, Kim N, Kwon M, Shon H, Kyoung Seo E, Kim HS, Young Lee S, Jeong W. Euphorbia factor L1 inhibits osteoclastogenesis by regulating cellular redox status and induces Fas-mediated apoptosis in osteoclast. Free Radic Biol Med. 2017; 112:191–9. 10.1016/j.freeradbiomed.2017.07.030 28774817

[14] Zhang JY, Lin MT, Yi T, Tang YN, Fan LL, He XC, Zhao ZZ, Chen HB. Apoptosis sensitization by Euphorbia factor L1 in ABCB1-mediated multidrug resistant K562/ADR cells. Molecules. 2013; 18:12793–808. 10.3390/molecules181012793 24135937 PMC6270536

[15] Sendi H, Yazdimamaghani M, Hu M, Sultanpuram N, Wang J, Moody AS, McCabe E, Zhang J, Graboski A, Li L, Rojas JD, Dayton PA, Huang L, Wang AZ. Nanoparticle Delivery of miR-122 Inhibits Colorectal Cancer Liver Metastasis. Cancer Res. 2022; 82:105–13. 10.1158/0008-5472.CAN-21-2269 34753773 PMC8732321

[16] Arzi L, Farahi A, Jafarzadeh N, Riazi G, Sadeghizadeh M, Hoshyar R. Inhibitory Effect of Crocin on Metastasis of Triple-Negative Breast Cancer by Interfering with Wnt/β-Catenin Pathway in Murine Model. DNA Cell Biol. 2018; 37:1068–75. 10.1089/dna.2018.4351 30351203

[17] Luo KW, Ko CH, Yue GG, Lee JK, Li KK, Lee M, Li G, Fung KP, Leung PC, Lau CB. Green tea (Camellia sinensis) extract inhibits both the metastasis and osteolytic components of mammary cancer 4T1 lesions in mice. J Nutr Biochem. 2014; 25:395–403. 10.1016/j.jnutbio.2013.11.013 24561153

[18] Zhou T, Zhang A, Kuang G, Gong X, Jiang R, Lin D, Li J, Li H, Zhang X, Wan J, Li H. Baicalin inhibits the metastasis of highly aggressive breast cancer cells by reversing epithelial-to-mesenchymal transition by targeting β-catenin signaling. Oncol Rep. 2017; 38:3599–607. 10.3892/or.2017.6011 29039569

[19] Song X, Zhou X, Qin Y, Yang J, Wang Y, Sun Z, Yu K, Zhang S, Liu S. Emodin inhibits epithelial-mesenchymal transition and metastasis of triple negative breast cancer via antagonism of CC-chemokine ligand 5 secreted from adipocytes. Int J Mol Med. 2018; 42:579–88. 10.3892/ijmm.2018.3638 29693154

[20] Fujii SI, Shimizu K. Immune Networks and Therapeutic Targeting of iNKT Cells in Cancer. Trends Immunol. 2019; 40:984–97. 10.1016/j.it.2019.09.008 31676264

[21] Dumauthioz N, Labiano S, Romero P. Tumor Resident Memory T Cells: New Players in Immune Surveillance and Therapy. Front Immunol. 2018; 9:2076. 10.3389/fimmu.2018.02076 30258445 PMC6143788

[22] Xie Q, Ding J, Chen Y. Role of CD8^+^ T lymphocyte cells: Interplay with stromal cells in tumor microenvironment. Acta Pharm Sin B. 2021; 11:1365–78. 10.1016/j.apsb.2021.03.027 34221857 PMC8245853

[23] Wu SY, Fu T, Jiang YZ, Shao ZM. Natural killer cells in cancer biology and therapy. Mol Cancer. 2020; 19:120. 10.1186/s12943-020-01238-x 32762681 PMC7409673

[24] Dong C. Cytokine Regulation and Function in T Cells. Annu Rev Immunol. 2021; 39:51–76. 10.1146/annurev-immunol-061020-053702 33428453

[25] Shaver KA, Croom-Perez TJ, Copik AJ. Natural Killer Cells: The Linchpin for Successful Cancer Immunotherapy. Front Immunol. 2021; 12:679117. 10.3389/fimmu.2021.679117 33995422 PMC8115550

[26] Zander R, Schauder D, Xin G, Nguyen C, Wu X, Zajac A, Cui W. CD4^+^ T Cell Help Is Required for the Formation of a Cytolytic CD8^+^ T Cell Subset that Protects against Chronic Infection and Cancer. Immunity. 2019; 51:1028–42.e4. 10.1016/j.immuni.2019.10.009 31810883 PMC6929322

[27] Moreno Ayala MA, Li Z, DuPage M. Treg programming and therapeutic reprogramming in cancer. Immunology. 2019; 157:198–209. 10.1111/imm.13058 30866047 PMC6587317

[28] Yu J, Green MD, Li S, Sun Y, Journey SN, Choi JE, Rizvi SM, Qin A, Waninger JJ, Lang X, Chopra Z, El Naqa I, Zhou J, et al. Liver metastasis restrains immunotherapy efficacy via macrophage-mediated T cell elimination. Nat Med. 2021; 27:152–64. 10.1038/s41591-020-1131-x 33398162 PMC8095049

[29] Ji D, Song C, Li Y, Xia J, Wu Y, Jia J, Cui X, Yu S, Gu J. Combination of radiotherapy and suppression of Tregs enhances abscopal antitumor effect and inhibits metastasis in rectal cancer. J Immunother Cancer. 2020; 8:e000826. 10.1136/jitc-2020-000826 33106387 PMC7592256

[30] Deng J, Kang Y, Cheng CC, Li X, Dai B, Katz MH, Men T, Kim MP, Koay EA, Huang H, Brekken RA, Fleming JB. DDR1-induced neutrophil extracellular traps drive pancreatic cancer metastasis. JCI Insight. 2021; 6:e146133. 10.1172/jci.insight.146133 34237033 PMC8492346

[31] Dai W, Liu S, Wang S, Zhao L, Yang X, Zhou J, Wang Y, Zhang J, Zhang P, Ding K, Li Y, Pan J. Activation of transmembrane receptor tyrosine kinase DDR1-STAT3 cascade by extracellular matrix remodeling promotes liver metastatic colonization in uveal melanoma. Signal Transduct Target Ther. 2021; 6:176. 10.1038/s41392-021-00563-x 33976105 PMC8113510

[32] Chen LY, Zhi Z, Wang L, Zhao YY, Deng M, Liu YH, Qin Y, Tian MM, Liu Y, Shen T, Sun LN, Li JM. NSD2 circular RNA promotes metastasis of colorectal cancer by targeting miR-199b-5p-mediated DDR1 and JAG1 signalling. J Pathol. 2019; 248:103–15. 10.1002/path.5238 30666650

[33] Shariati M, Evans KW, Zheng X, Bristow CA, Ng PK, Rizvi YQ, Tapia C, Yang F, Carugo A, Heffernan TP, Peoples MD, Tripathy D, Meric-Bernstam F. Combined inhibition of DDR1 and CDK4/6 induces synergistic effects in ER-positive, HER2-negative breast cancer with PIK3CA/AKT1 mutations. Oncogene. 2021; 40:4425–39. 10.1038/s41388-021-01819-0 34108622 PMC13101480

[34] Vella V, Giuliano M, Nicolosi ML, Majorana MG, Marć MA, Muoio MG, Morrione A, Maggiolini M, Lappano R, De Francesco EM, Belfiore A. DDR1 Affects Metabolic Reprogramming in Breast Cancer Cells by Cross-Talking to the Insulin/IGF System. Biomolecules. 2021; 11:926. 10.3390/biom11070926 34206590 PMC8301864

[35] Lim HI, Yamamoto J, Han Q, Sun YU, Nishino H, Tashiro Y, Sugisawa N, Tan Y, Choi HJ, Nam SJ, Bouvet M, Hoffman RM. Response of Triple-negative Breast Cancer Liver Metastasis to Oral Recombinant Methioninase in a Patient-derived Orthotopic Xenograft (PDOX) Model. In Vivo. 2020; 34:3163–9. 10.21873/invivo.12151 33144420 PMC7811664

[36] Wang Y, Liu S, Liu H, Li W, Lin F, Jiang L, Li X, Xu P, Zhang L, Zhao L, Cao Y, Kang J, Yang J, et al. SARS-CoV-2 infection of the liver directly contributes to hepatic impairment in patients with COVID-19. J Hepatol. 2020; 73:807–16. 10.1016/j.jhep.2020.05.002 32437830 PMC7211738

[37] Johnstone CN, Smith YE, Cao Y, Burrows AD, Cross RS, Ling X, Redvers RP, Doherty JP, Eckhardt BL, Natoli AL, Restall CM, Lucas E, Pearson HB, et al. Functional and molecular characterisation of EO771.LMB tumours, a new C57BL/6-mouse-derived model of spontaneously metastatic mammary cancer. Dis Model Mech. 2015; 8:237–51. 10.1242/dmm.017830 25633981 PMC4348562

[38] Naiman S, Huynh FK, Gil R, Glick Y, Shahar Y, Touitou N, Nahum L, Avivi MY, Roichman A, Kanfi Y, Gertler AA, Doniger T, Ilkayeva OR, et al. SIRT6 Promotes Hepatic Beta-Oxidation via Activation of PPARα. Cell Rep. 2019; 29:4127–43.e8. 10.1016/j.celrep.2019.11.067 31851938 PMC7165364

