# Supplementary material for: Euphorbia factor L1 suppresses breast cancer liver metastasis via DDR1-mediated immune infiltration
Source: Aging (Albany NY). 2023 Sep 14;15(17):9217–29. doi: 10.18632/aging.205030 (PMC10522367; doi:10.18632/aging.205030)
Supplement: Supplementary Materials [file aging-15-205030-s001.pdf]

SUPPLEMENTARY MATERIALS

Establishment of *in situ* model of breast cancer

200 μL of 4T1-LUC cell suspension at a concentration of 2.5×10^7/mL was injected subcutaneously into the back of the scapula of female BALB/c mice. Drug interventions (EFL1 50 mg/kg, daily gavage; doxorubicin, 5 mg/kg, tri-weekly intraperitoneal injection) were given at 7 days after tumor implantation for a total of 2 weeks. An AniView600 animal imaging system was applied to monitor the metastasis of tumor cells in mice after 7 and 14 days of drug administration. On the second day of the last administration, all mice were sacrificed and sampling was conducted.

Preparation of EFL1 containing serum

Female BALB/c mice were treated with EFL1 (25, 50 mg/kg/day) by gavage for 10 days. Two hours after the last administration, the blood was collected from the retrobulbar venous plexus and serum was extracted by centrifugation. The serum was then inactivated by heating in a 56°C water bath for 30 min, then stored at −20°C for future use.

CD3+ T cell sorting

(1) Resuspend the single-cell suspension of mouse tumor tissue in PEB solution at a concentration of 10^7–10^8 cells/mL.
(2) Block with FcR blocking reagent at 4°C for 10 min.
(3) Add 10 uL of CD3 antibody magnetic bead complex to every 100 μL of cells, and incubate at room temperature for 15 min in the dark.
(4) Resuspend the cells co-incubated with magnetic beads in 1 mL PEB solution.
(5) Put the MS Separation column on the matching magnetic stand, and add the cell suspension for adsorption.
(6) Wash the column with PEB solution until the effluent is clear. Remove unadsorbed negative cells.
(7) Add 1mL of PEB solution to the column, remove the column from the magnetic stand, use the matching syringe to push out the cells, and collect CD3 positive T cells for subsequent experiments.

Detection of migration ability of 4T1 cells co-cultured with CD3+ T cells

(1) Plant 4T1 cells in a transwell chamber at 1×10^5 cells/well. Medium volume, 100 μL.
(2) Put the cell-added chamber into a 24-well plate seeded with CD3+ T cells, and incubate for 48 h with or without EFL1-containing serum.
(3) Take out the chamber and wash it with PBS;
(4) Add 4% paraformaldehyde to fix 4T1 cells for 15 min at room temperature.
(5) Wash with PBS 3 times.
(6) Add crystal violet staining solution for 30 min at room temperature.
(7) Discard the crystal violet staining solution and wash 3 times with PBS.
(8) Gently wipe off the inner cells of the transwell chamber with a cotton ball.
(9) Observe the tumor cells migrating to the other side of the chamber under a microscope.
