# Supplementary material for: Euphorbia factor L1 suppresses breast cancer liver metastasis via DDR1-mediated immune infiltration
Source: Aging (Albany NY). 2023 Sep 14;15(17):9217–29. doi: 10.18632/aging.205030 (PMC10522367; doi:10.18632/aging.205030)
Supplement: Supplementary Figures [file aging-15-205030-s002.pdf]

SUPPLEMENTARY FIGURES

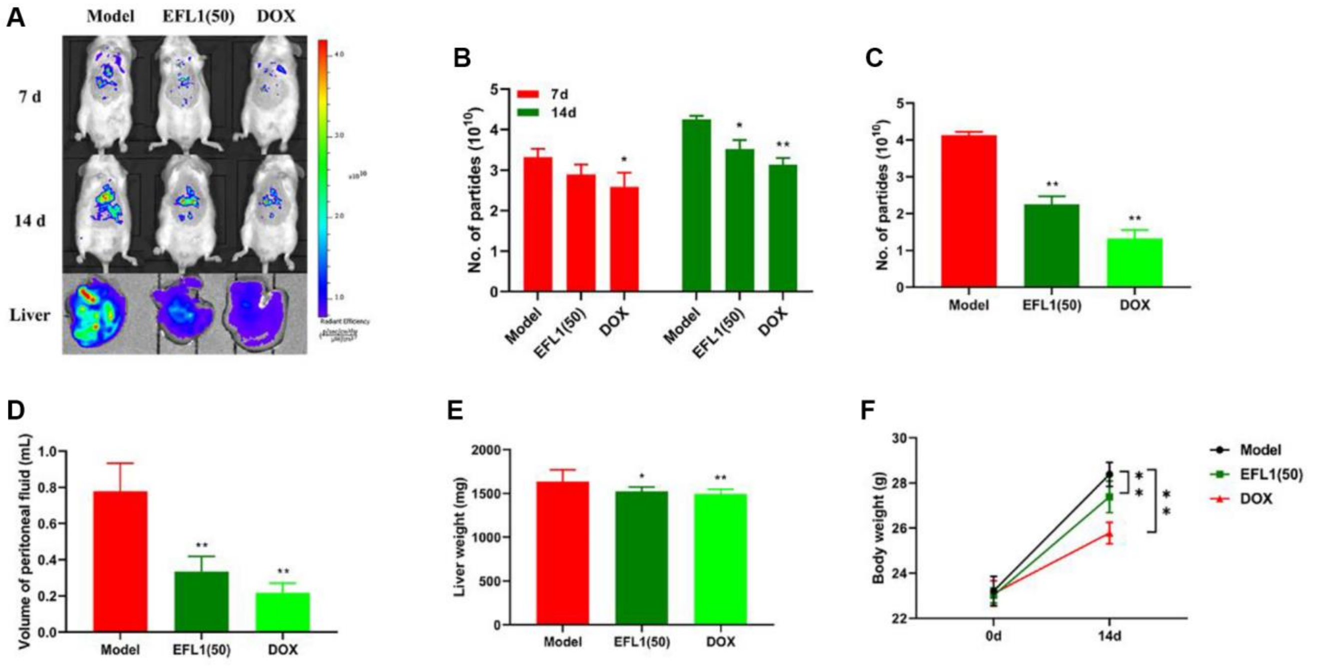

Supplementary Figure 1. EFL1 alleviates liver metastasis of *in situ* breast cancer. Treatment with EFL1 or DOX for 2 weeks significantly decreased metastatic luciferase signals (**A**–**C**, $n = 3$), peritoneal fluid (**D**, $n = 9$), liver weight (**E**, $n = 9$) and body weight (**F**, $n = 9$) of mice with 4T1-LUC breast cancer. Data were compared using two-way ANOVA with Tukey's post hoc (**B**, **F**) or one-way ANOVA with Dunnett's post hoc (**C**–**E**). $^{**}P < 0.01$. Abbreviations: ns: not significant. $^{*}P < 0.05$, $^{**}P < 0.01$ vs. Model.

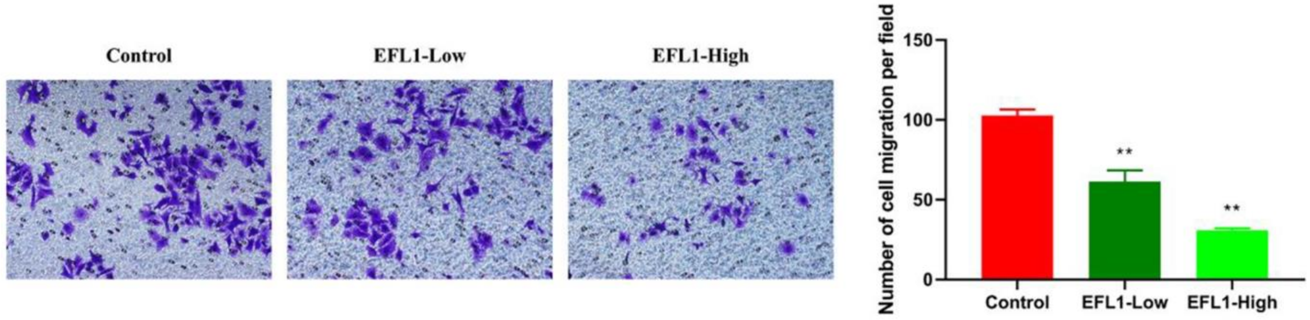

Supplementary Figure 2. EFL1 decreases the migration of 4T1 cells co-cultured with CD3+ T cells. 4T1 cells were co-cultured with CD3+ T cells for 48 h with or without EFL1-containing serum. Cell mitigation was observed under a microscope. Treatment with EFL1-containing serum greatly decreased the number of mitigating 4T1 cells. $n = 3$. Data were compared using one-way ANOVA with Dunnett's post hoc. $^{**}P < 0.01$ vs. Control.
